# Dual Aetiology of Lower Limb Claudication: A Rare Case of Coexisting Lumbar Facet Joint Synovial Cyst and Critical Lower Limb Arterial Stenoses

**DOI:** 10.7759/cureus.92832

**Published:** 2025-09-21

**Authors:** Claire Ng, Adrian Ng, Keh Oon Ong, Moorthy Sinnasamy

**Affiliations:** 1 Radiology, Aberdeen Royal Infirmary, Aberdeen, GBR; 2 Radiology, Salford Royal Hospital, Northern Care Alliance NHS Foundation Trust, Salford, GBR; 3 Radiology, Gleneagles Hospital Kuala Lumpur, Kuala Lumpur, MYS

**Keywords:** critical limb ischaemia, facet joint cyst rupture, interventional radiology, lower-extremity claudication, lumbar facet joint cyst, neurogenic claudication, percutaneous transluminal angioplasty, vascular claudication

## Abstract

Lower limb claudication is a common symptom with diverse aetiologies, most frequently neurogenic or vascular in origin. Identifying and discerning between these causes is essential, especially in patients with cardiovascular risk factors, as misdiagnosis may lead to suboptimal treatment. Dual pathology, though rare, can pose a significant diagnostic challenge. We report a case of a 60-year-old male farmer with a history of coronary artery disease who presented with progressive bilateral lower limb claudication and lumbosacral back pain. MRI spine revealed a right L4-L5 facet joint synovial cyst causing central spinal canal stenosis, while CT angiography demonstrated bilateral critical femoral artery stenoses. He underwent a percutaneous CT-guided facet joint synovial cyst rupture, which provided partial symptom relief, mainly from neurogenic claudication. Despite this, he still experienced persistent exertional claudication. He subsequently underwent staged endovascular revascularisation of the femoral arteries under Interventional Radiology, resulting in complete resolution of symptoms. At the three-month follow-up, he remained asymptomatic and had returned to his baseline physical function. This case highlights the diagnostic challenges associated with claudication when neurogenic and vascular features overlap and demonstrates that although the coexistence of neurogenic and vascular claudication is uncommon, it should be considered in patients presenting with atypical or refractory symptoms. In this particular case, a sequential percutaneous approach, addressing the spinal pathology first, followed by vascular insufficiency, resulted in excellent functional outcomes. These findings emphasise the importance of thorough clinical assessment and appropriate imaging in identifying the aetiology, thus preventing misdiagnosis. In addition, it also demonstrates that staged, minimally invasive radiological interventions can obviate the need for open surgery whilst still achieving complete symptom resolution and restoration of function.

## Introduction

Lower limb claudication is a common clinical complaint, usually attributed to either neurogenic compression secondary to lumbar spinal canal stenosis, which affects 11% of the population [1], or vascular insufficiency secondary to peripheral arterial disease. 

Vascular claudication arises from impaired blood supply to exercising muscles, typically manifesting as reproducible calf pain on exertion that resolves with rest [2]. In contrast, neurogenic claudication results from compression of the cauda equina or spinal nerve roots, frequently caused by degenerative changes such as facet joint hypertrophy, ligamentum flavum thickening, or disc protrusion [1,3]. Symptoms often improve with spinal flexion, sitting, or squatting, distinguishing it from vascular disease [4,5].

Although both conditions are prevalent in the elderly, the simultaneous occurrence of critical vascular stenosis and neurogenic compression as independent contributors to claudication has been rarely documented. Such overlap presents a diagnostic challenge, as the clinical features may be misleading, leading to misdiagnosis, incomplete treatment, or unnecessary interventions. The potential for anchoring bias, attributing all symptoms to one presumed aetiology, further complicates evaluation. Hence, discerning between these aetiologies is critical [3], yet they may coexist, particularly in older patients with cardiovascular risk factors. 

Imaging plays a pivotal role in resolving this diagnostic dilemma. MRI of the lumbar spine is the gold standard for assessing degenerative spinal pathology, including the less common but clinically significant lumbar facet joint synovial cysts (LFSCs) [6,7]. These cysts arise from the facet joint capsule and may impinge on adjacent neural structures, producing neurogenic claudication [8-10]. Concurrently, CT angiography provides high-resolution vascular mapping, allowing accurate characterisation of arterial stenoses in the iliac and femoropopliteal segments [11].

We present the case of a 60-year-old man with extensive cardiovascular risk factors who developed severe bilateral claudication due to a dual pathology: a lumbar facet joint cyst causing spinal canal stenosis and bilateral critical lower limb artery stenoses. Sequential, targeted management of both conditions, first with CT-guided lumbar facet joint cyst injection and subsequently with percutaneous transluminal angioplasty, resulted in complete symptom resolution. This case underscores the value of a comprehensive diagnostic approach in claudication and highlights the role of radiological investigations in guiding stepwise treatment.

## Case presentation

A 60-year-old male farmer presented with persistent lumbosacral back pain and progressive bilateral lower limb claudication. His past medical history includes percutaneous coronary intervention (PCI) four years prior to presentation for circumflex artery stenosis. He also had several cardiovascular risk factors, such as an extensive smoking history of 42 pack-years, high alcohol consumption, and dyslipidemia. His symptoms began after walking approximately 50 meters and were described as lower back pain associated with numbness and a burning, aching pain down the bilateral gluteal regions, thighs, and legs when standing. These symptoms worsened markedly with ambulation. He reported temporary relief when sitting or squatting. Analgesics, namely paracetamol and celecoxib, provided minimal relief, and his symptoms were so severe that he had to discontinue his farming activities. 

**Figure 1:**
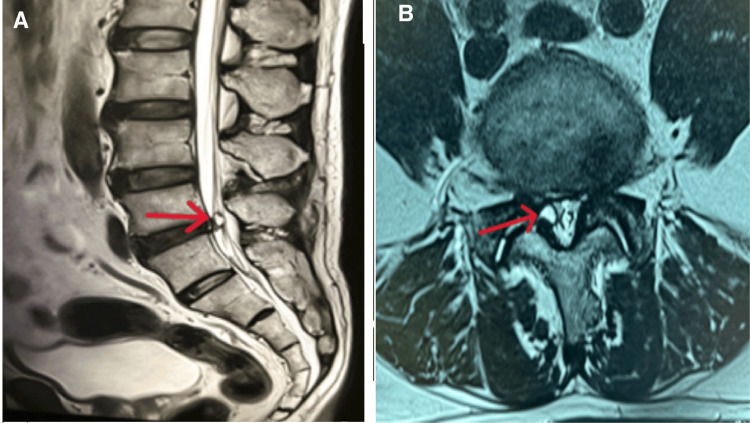
T2-weighted MRI images (A) Sagittal view of the lumbosacral region demonstrating a lumbar facet joint synovial cyst measuring 4.1 x 4.3 x 6.5 mm (red arrow) within the spinal canal. (B) Axial view of the lumbar region at L4/L5 vertebral level demonstrating the cyst (red arrow) lying anteromedially to the degenerated right L4/L5 facet joint, causing severe spinal canal stenosis and exit foraminal stenosis.

**Figure 2:**
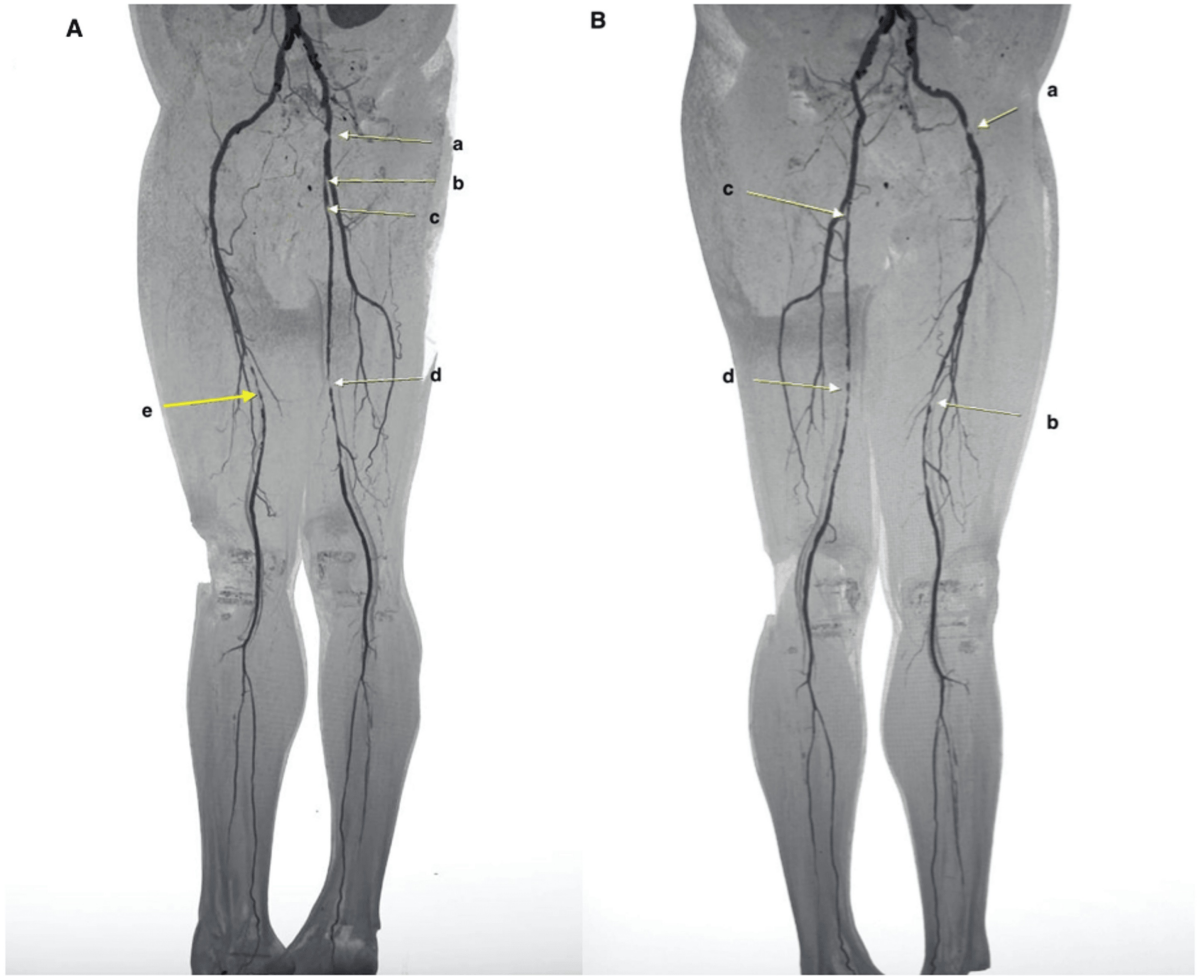
CT angiogram of the lower limb arteries; yellow arrows indicate regions of arterial stenosis (A) First oblique view of CT angiogram showing stenosis in the right CFA (a), ostium of right SFA (b), proximal right SFA (c), mid-distal right SFA (d), and mid-distal left SFA (e). (B) Second oblique view of CT angiogram showing stenosis in the right CFA (a), mid-distal right SFA (b), proximal left SFA (c), and mid-distal left SFA (d). CFA: common femoral artery; SFA: superficial femoral artery

On examination, distal pulses were weak or absent bilaterally. The right femoral pulse was very faint, and the left femoral pulse was normal. Bilateral popliteal pulses and posterior tibialis pulses were faint. Bilateral dorsalis pedis pulses were absent. Neurological examination revealed no focal deficits. Due to lack of response to conservative measures over a period of several months, he underwent an MRI of the lumbar spine, which revealed a synovial cyst arising from the right L4-L5 facet joint, causing significant compression of the thecal sac resulting in central canal stenosis (Figure 1). Additionally, CT angiogram of the lower limb vasculature revealed bilateral multi-level critical stenoses of the femoral arteries (Figure 2).

**Figure 3:**
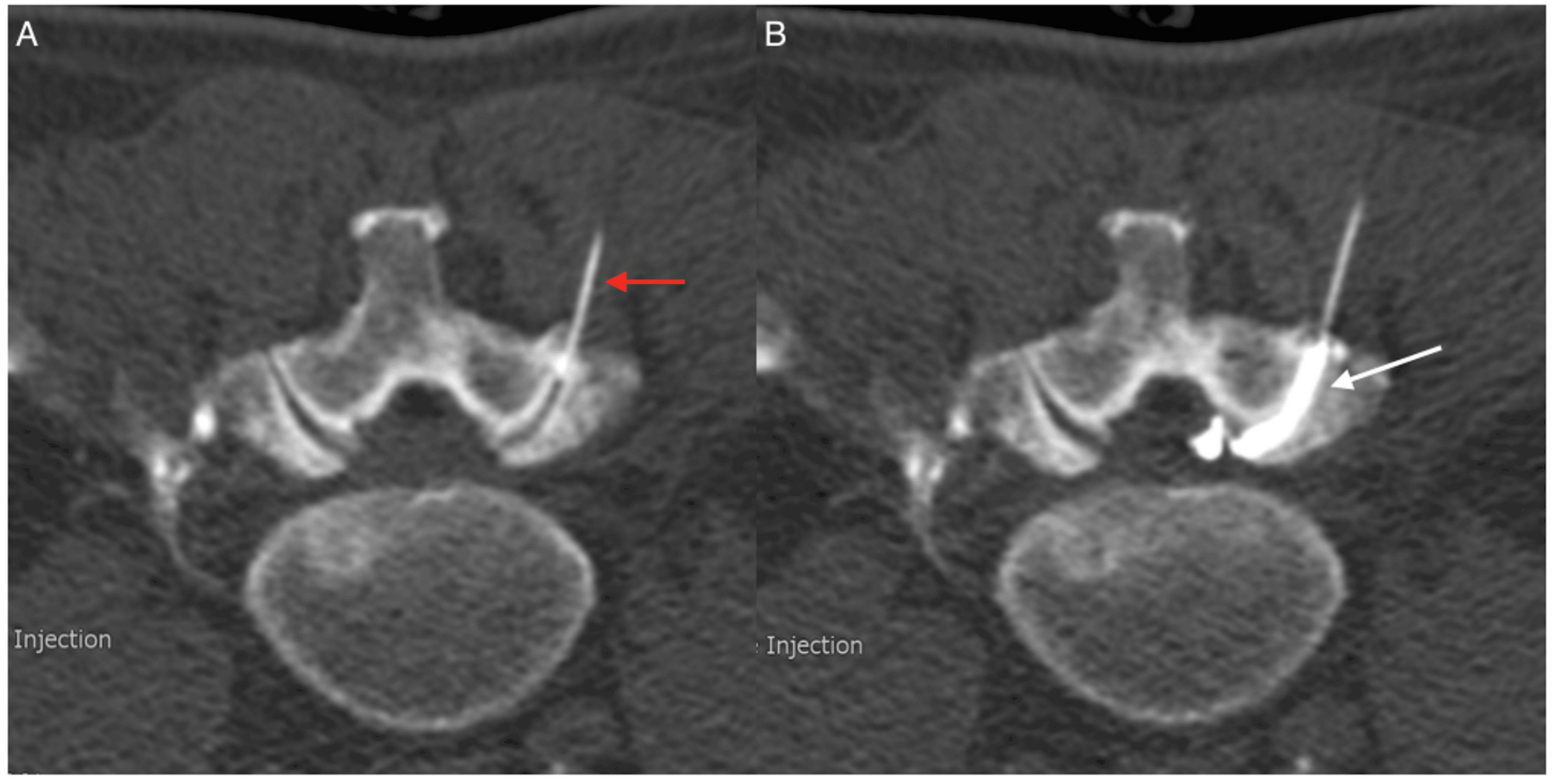
Axial CT images (A) A spinal needle (red arrow) is inserted into the right L4/L5 facet joint and contrast is injected through the right L4/L5 facet joint. (B) The contrast fills the cyst and causes its rupture (white arrow).

A CT-guided percutaneous injection of the lumbar facet joint synovial cyst (Figure 3) with contrast, then subsequently with corticosteroid and local anaesthetic, resulted in partial symptom relief, specifically the burning and heaviness sensation in the lower back and gluteal region that occurred with standing. However, his exertional, distance-limited claudication did not resolve with the spinal intervention; instead, it persisted, with symptoms worsening in the right lower limb. 

**Figure 4:**
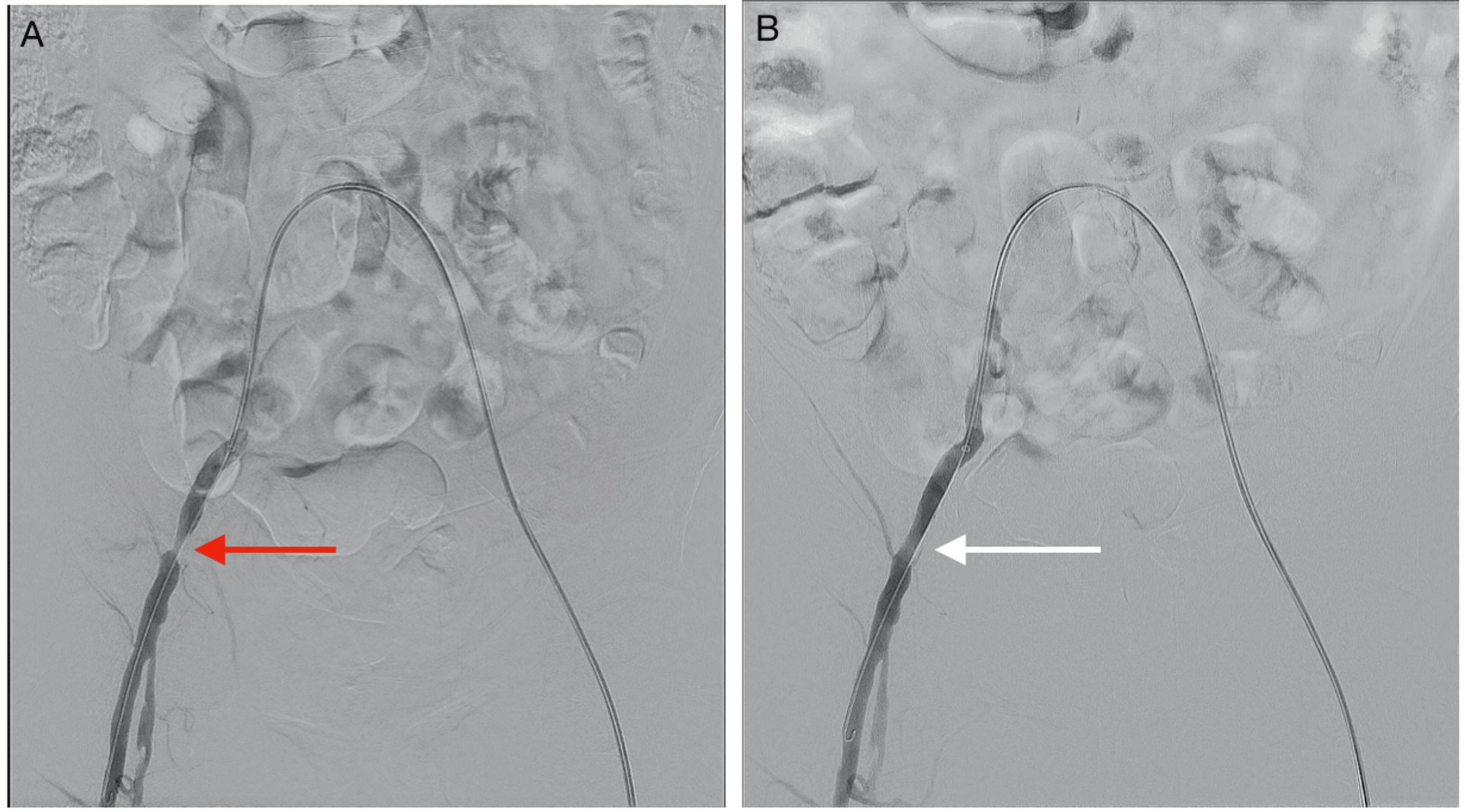
DSA images showing the right common femoral artery during endovascular intervention (A) Severe stenosis of the right CFA (indicated by the red arrow) before PTA. (B) Increase in vessel calibre of the right CFA (indicated by the white arrow) post PTA. DSA: digital subtraction angiography; CFA: common femoral artery; PTA: percutaneous transluminal angioplasty

**Figure 5:**
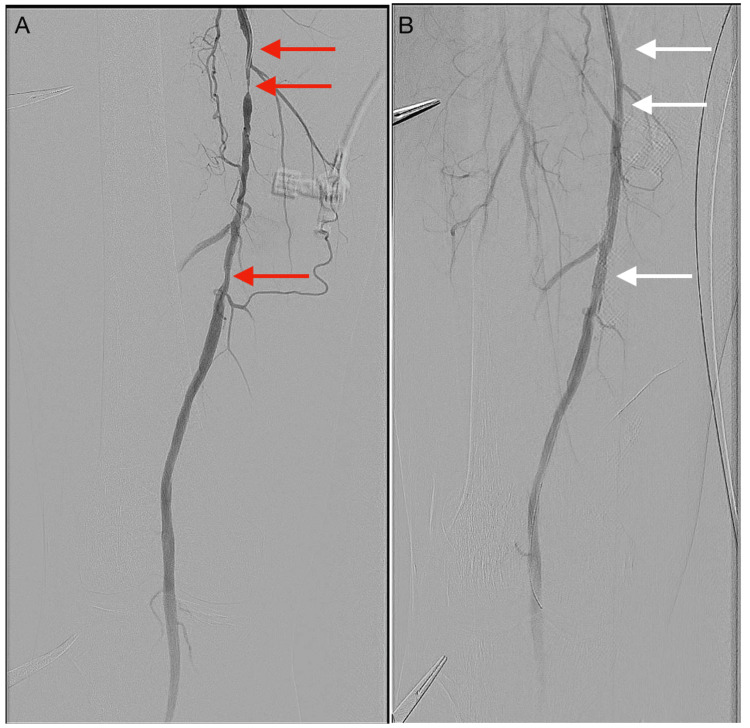
DSA images showing the mid and distal right superficial femoral artery during endovascular intervention (A) Diffuse disease of the mid and distal right SFA (red arrows) before PTA. (B) Increase in vessel calibre of the mid and distal right SFA (white arrows) post PTA. DSA: digital subtraction angiography; SFA: superficial femoral artery; PTA: percutaneous transluminal angioplasty

**Figure 6:**
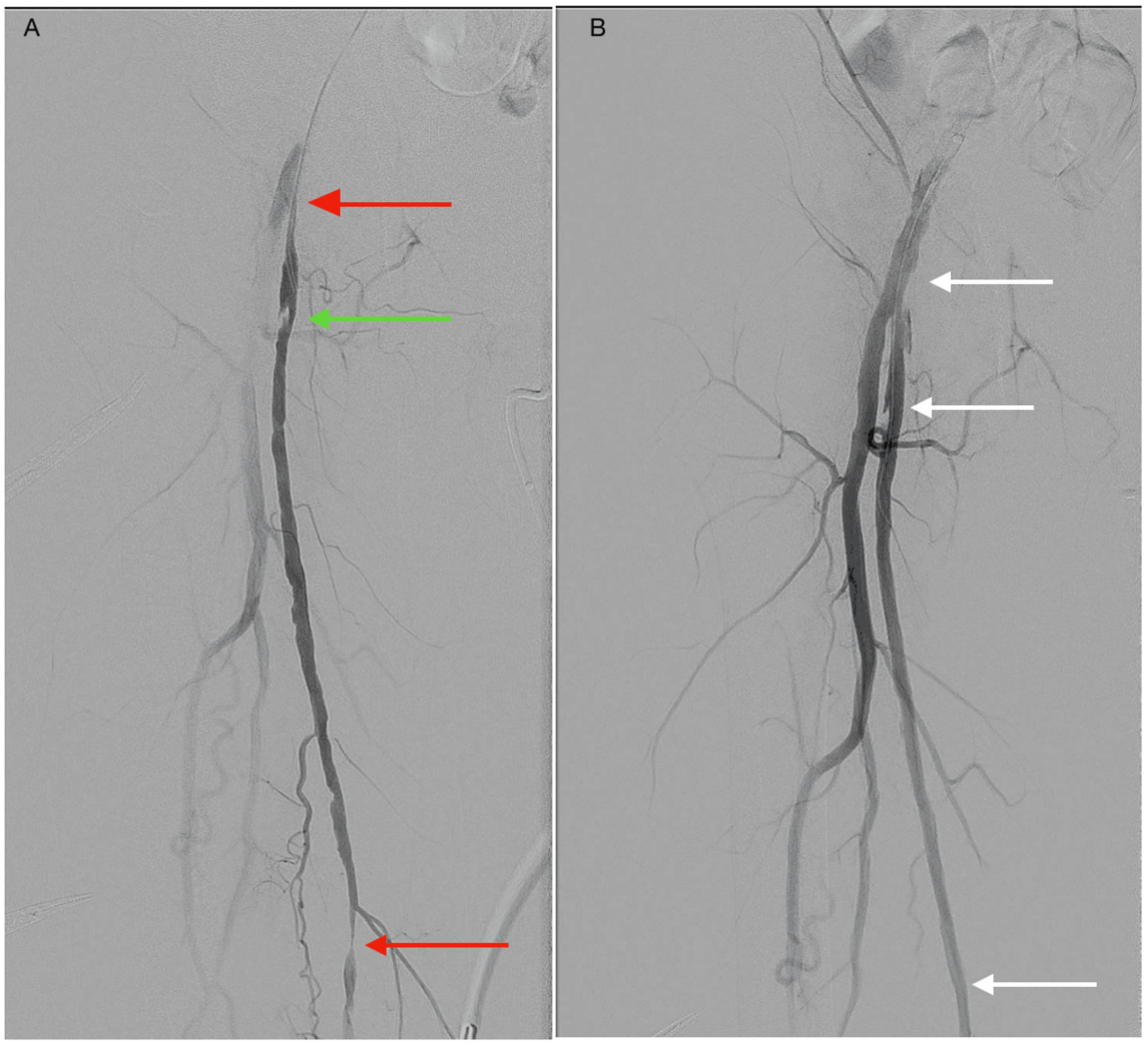
DSA images demonstrating the proximal and middle segment of the right superficial femoral artery during endovascular intervention (A) Severe ostial stenosis of the right SFA and severe stenosis of the mid-right SFA are indicated by red arrows, a complex ulcerated plaque is indicated by a green arrow. (B) The affected stenosed segments were successfully treated by PTA (white arrows). DSA: digital subtraction angiography; SFA: superficial femoral artery; PTA: percutaneous transluminal angioplasty

**Figure 7:**
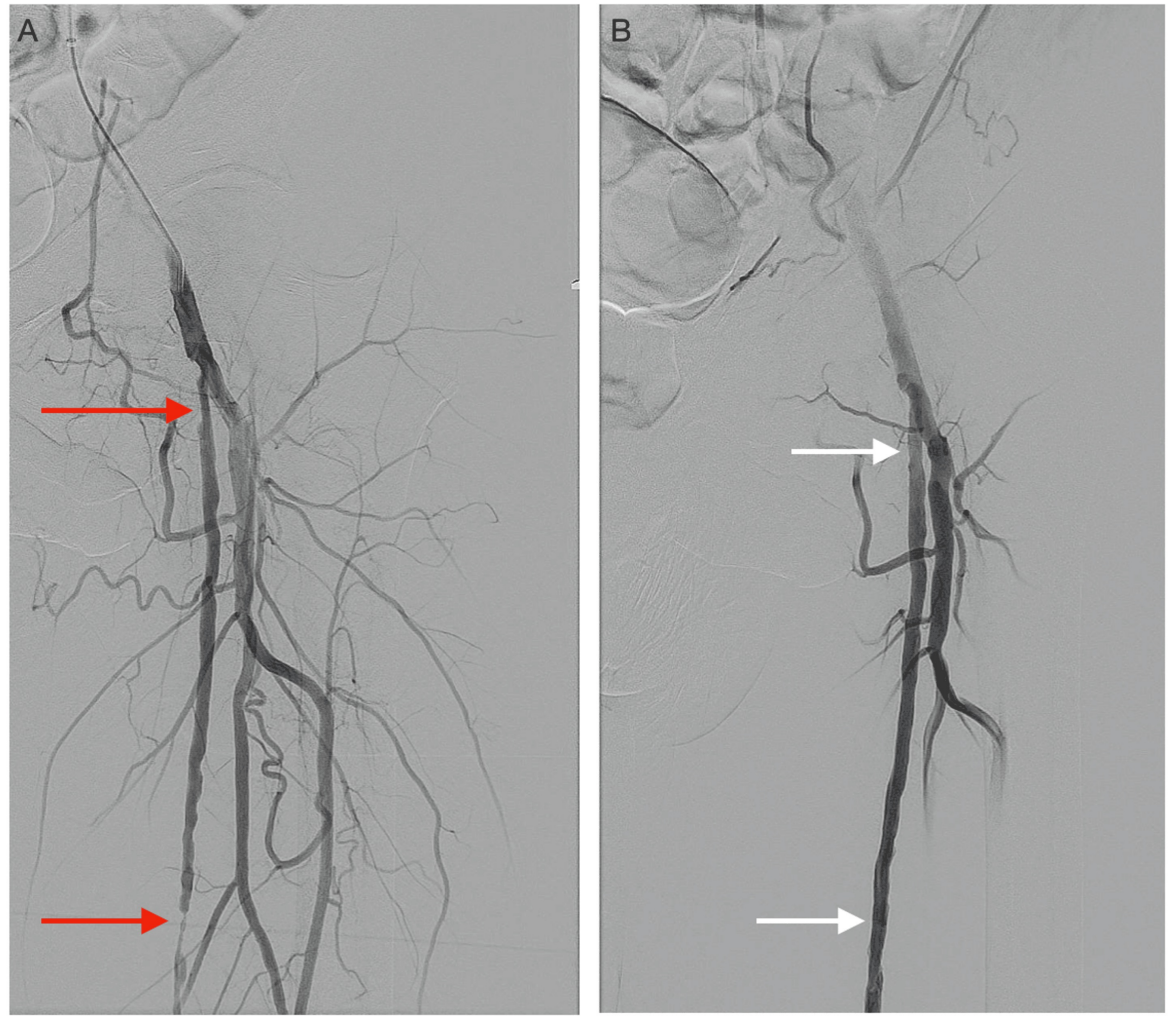
DSA images demonstrating the proximal to the middle segment of the left superficial femoral artery during endovascular intervention (A) Severe stenosis of the proximal left SFA and mid-left SFA (red arrows) before PTA. (B) The regions of stenosis were successfully treated by PTA (white arrows). DSA: digital subtraction angiography; SFA: superficial femoral artery; PTA: percutaneous transluminal angioplasty

**Figure 8:**
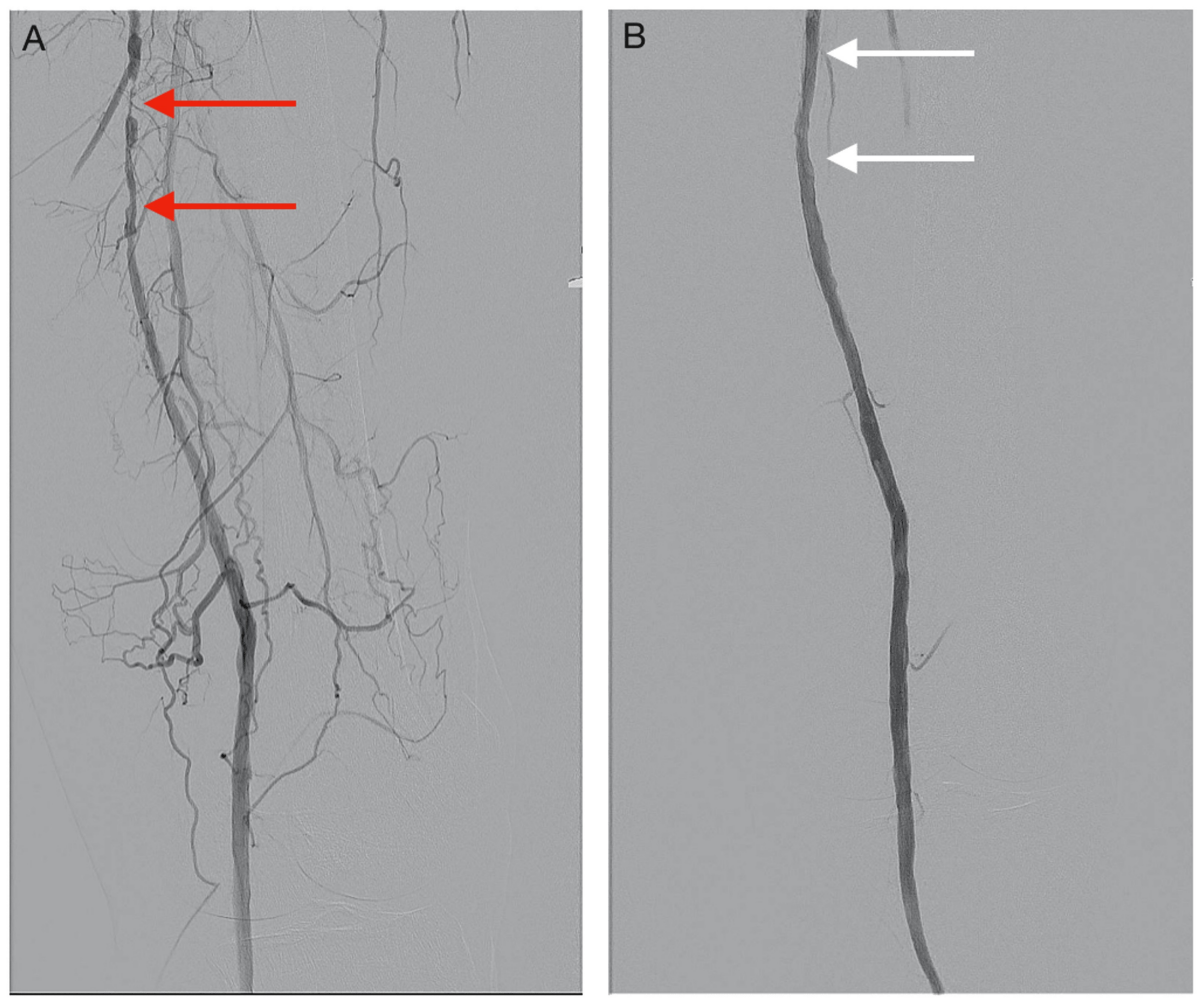
DSA images demonstrating the mid to distal left superficial femoral artery during endovascular intervention (A) Severe stenosis of the mid to distal left SFA (red arrows) before PTA. (B) The regions of stenosis were successfully treated (white arrows) post PTA. DSA: digital subtraction angiography; SFA: superficial femoral artery; PTA: percutaneous transluminal angioplasty

Weeks later, the patient underwent sequential percutaneous transluminal angioplasties to address the critical bilateral lower limb stenoses. The first procedure targeted the right common and superficial femoral artery (Figures 4-6). This improved flow in these arteries, thus improving his walking distance marginally and completely resolving the ipsilateral calf claudication. A second angioplasty was then performed on the left superficial femoral artery (Figures 7-8). This finally fully resolved his claudication symptoms. Post procedure, he reported full resolution of symptoms and was able to ambulate over 500 meters without discomfort. At the three-month follow-up, he remained asymptomatic and had resumed his farming activities.

## Discussion

Differentiating neurogenic from vascular claudication is crucial but challenging. Neurogenic claudication often improves with spinal flexion [4], such as when adopting a stooped posture when pushing a shopping cart, cycling, or squatting, as this increases the space within the spinal canal. In contrast, it worsens with spinal extension and loading [5], such as during standing, as the space within the spinal canal decreases. Vascular claudication is reliably induced by physical exertion and relieved by rest. In this patient, overlapping features masked the dual pathology. 

Degenerative spinal stenosis arises when age-related degeneration of the intervertebral disc bulges into the central spinal canal [1,3]. This is often associated with osteophytes of the vertebral end plate. The loss of disc height imposes increased mechanical stress upon the facet joints and the ligamentum flavum, leading to hypertrophy and inward buckling of the latter into the spinal canal. In addition, the facet joints undergo degenerative changes, with hypertrophy of the joints and development of osteophytes impinging upon the lateral recesses [3]. In the most advanced cases, development of a facet joint synovial cyst will further contribute to the neurogenic component by compressing adjacent nerve roots [8-10]. 

LFSCs are fluid-filled outpouches lined with synovium that arise from the facet joint capsule. They are found most often at the L4-L5 level in the setting of degenerative facet arthropathy. On imaging, they appear as well-circumscribed masses in direct contact with or situated immediately adjacent to the facet joint [8-10].

In terms of treating the LFSC, in two retrospective analyses, CT-guided LFSC injection achieved effectiveness rates of 56.3% (36/64 patients) and 66.1% (80/121 patients), respectively [12,13]. At long-term follow-up, these patients did not require surgical intervention, as the CT-guided procedure provided sustained symptomatic relief. No postoperative complications were reported in all cases.

There is limited evidence on the efficacy of surgery for spinal stenosis [14]. Even in the best centres, decompressive laminectomy only offered symptomatic relief in up to 80% patients, with pain relapsing in one-third of patients 7-10 years later [15]. Surgical intervention also carries nontrivial complications such as dural tear, CSF leak, deep vein thrombosis, postoperative infection, haemorrhage, non-union following fusion, and worsening postoperative stability (in particular post-laminectomy without fusion) [16,17].

In terms of managing the peripheral arterial disease, sequential angioplasty in a staged manner, rather than simultaneous bilateral intervention, is often preferred in stable patients as it reduces contrast load and renal risk (especially in those with cardiovascular disease) [18], limits cumulative procedural time, reduces the risk of complications, and allows clear assessment of symptom improvement following unilateral revascularization. In addition, doing the procedures in a staged manner in the current study was also due to the annual financial limit imposed by the patient's private health insurer, which precluded simultaneous bilateral PTA within the same session.

This case illustrates the clinical importance of not anchoring on a single diagnosis when multiple pathologies may coexist. It also highlights the role of imaging, multidisciplinary assessment, and stepwise management in resolving complex claudication presentations. Early suspicion and identification of dual pathology can avoid unnecessary surgical intervention (e.g., spinal decompression) when vascular insufficiency is also present and correctable via endovascular therapy.

Few reports have described this dual-pathology of concurrent spinal and vascular causes of claudication treated successfully with an entirely percutaneous approach. With an increasingly aging population and overlapping risk factors for spinal degeneration and atherosclerosis, clinicians must maintain vigilance for coexisting aetiologies in patients with atypical or treatment-resistant claudication.

## Conclusions

Lower limb claudication can stem from neurogenic or vascular causes, but concurrent pathology is uncommon and diagnostically challenging. Recognising and treating both aetiologies is crucial for full symptom resolution. The sequence of treatment intervention would depend on the clinical features and the patient's main presenting complaint. In this case, the main complaint was severe lower back pain radiating to the bilateral gluteal region on minimal ambulation, which caused the patient to stop farming. Hence, the priority was to treat the LFSC first. After the initial percutaneous CT-guided LFSC injection, which helped to relieve him of his neurogenic pain, he was still left with vascular claudication pain in both calves. This was subsequently treated with percutaneous transluminal angioplasty.

This case highlights the need to adopt a systematic diagnostic approach to claudication, especially with the possibility of a dual aetiology. In our patient, sequential treatment, first targeting the LFSC, then the peripheral arterial disease, proved effective. With careful clinical evaluation and radiological correlation, a well-planned staged therapeutic intervention can lead to complete resolution of symptoms with full restoration of function, thus avoiding subjecting the patient to open surgery and its associated complications. 
